# Supplementary material for: Steady motion of 80-nm-size skyrmions in a 100-nm-wide track
Source: Nat Commun. 2024 Jul 4;15:5614. doi: 10.1038/s41467-024-49976-6 (PMC11224351; doi:10.1038/s41467-024-49976-6)
Supplement: Supplementary file 1 — Supplementary Information [file 41467_2024_49976_MOESM1_ESM.pdf]

Supplementary Information for

Steady motion of 80-nm-size skyrmions in a 100-nm-wide track

Dongsheng Song[1,2,*], Weiwei Wang[1,2], Shuisen Zhang[2,3], Yizhou Liu[2], Ning Wang[2], Fengshan Zheng[4], Mingliang Tian[2,5,6], Rafal E. Dunin-Borkowski[4], Jiadong Zang[7,8] and Haifeng Du[1,2,5,*]

[1]Institutes of Physical Science and Information Technology, Anhui University, Hefei 230601, China

[2]Anhui Province Key Laboratory of Low-Energy Quantum Materials and Devices, High Magnetic Field Laboratory, HFIPS, Chinese Academy of Sciences, Hefei, Anhui 230031, China

[3]University of Science and Technology of China, Hefei 230026, China

[4]Ernst Ruska-Centre for Microscopy and Spectroscopy with Electrons and Peter Grünberg Institute, Forschungszentrum Jülich, 52425 Jülich, Germany

[5]Science Island Branch of Graduate School, University of Science and Technology of China, Hefei, Anhui 230026, China

[6]School of Physics and Optoelectronic Engineering, Anhui University, Hefei, 230601, China

[7]Department of Physics and Astronomy, University of New Hampshire, Durham, New Hampshire 03824, USA

[8]Materials Science Program, University of New Hampshire, Durham, New Hampshire 03824, USA

*Corresponding author: dsong@ahu.edu.cn, duhf@hmfl.ac.cn

Supplementary Note I – Micromagnetic framework of skyrmion dynamics

In chiral magnets such as FeGe, the current flow will result in torques, which rotate the local magnetization. This process can be described by the Landau-Lifshitz-Gilbert (LLG) equation, including spin-transfer torques (Zhang-Li model), according to the expression[1]

$$\frac{\partial \mathbf{m}}{\partial t} = -\gamma \mathbf{m} \times \mathbf{H}_{\mathrm{eff}} + \alpha \mathbf{m} \times \frac{\partial \mathbf{m}}{\partial t} - (\mathbf{u} \cdot \nabla)\mathbf{m} + \beta[\mathbf{m} \times (\mathbf{u} \cdot \nabla)\mathbf{m}] \quad , \tag{1}$$

where $\mathbf{m}$ is a unit vector of the magnetization, γ is the gyromagnetic ratio, $\mathbf{H}_{\mathrm{eff}}$ is the total effective field and α is the Gilbert damping. The third and fourth terms on the right side of the equation are the spin-transfer torques. The parameter u is defined as

$$\mathbf{u} = -\frac{gP\mu_{\mathrm{B}}}{2eM_{\mathrm{s}}}\mathbf{j} \quad , \tag{2}$$

where $\mathbf{j}$ is the current density, g is the Landé factor, μ_{B} is the Bohr magneton, e (> 0) is the electron charge, P is the polarization rate of the current, M_{s} is the saturation magnetization and β is the nonadiabatic spin-transfer parameter[2].

We first use Thiele's approach to describe skyrmion dynamics in a nanotrack. We start with translational motion and ignore skyrmion distortions, *i.e.*, using a rigid skyrmion model such that $\mathbf{m}(\mathrm{r}, t) = \mathbf{m}(\mathrm{r} - \mathrm{R(t)})$, where R is the geometric center of the skyrmions. The Thiele equation takes the form[3–6]:

$$\mathbf{G} \times (\mathbf{u} - \mathbf{v}) + \mathcal{D}(\beta\mathbf{u} - \alpha\mathbf{v}) + \mathbf{F}^{\mathbf{pin}} + \mathbf{F} = 0 \quad , \tag{3}$$

where $\mathbf{G} = G\mathbf{e}_z$ and $G = 4\pi Q$ describes the Magnus force, $\mathbf{e}_z$ is a unit vector along the z-axis and Q is the skyrmion number. The second term denotes the dissipative force, where $\mathcal{D} = 4\pi\, \eta_{ij}$ is the tensor associated with the shape factor and $\eta_{ij} = (1/4\pi) \int (\partial_i \mathbf{m} \times \partial_j \mathbf{m})\, \mathrm{d}x\, \mathrm{d}y$. For a magnetic skyrmion without distortion, $\eta_{ij} = \delta_{ij}\eta$ and η is typically close to unity. The pinning force $\mathbf{F}^{\mathrm{pin}}$ originates from disorder in the material and is given by $\mathbf{F}_i^{\mathrm{pin}} = \int \gamma \mathbf{m} \cdot [\partial_i \mathbf{m} \times (\mathbf{m} \times \mathbf{H}_{\mathrm{pin}})]\, \mathrm{d}x\, \mathrm{d}y$. The environmental force can be defined as $\mathbf{F} = -\nabla \mathrm{U}$ and $U = U(\mathbf{r})$ is the phenomenological environmental potential representing the pushing force imposed by spin twisting at the edges. Within the framework of micromagnetic theory, the

phenomenological potential is directly connected to the total micromagnetic energy E, *i.e.*, $U = (\gamma/\mu_0 M_s L)E$, where L is the thickness of the sample.

We assume that currents are applied in the x-direction, *i.e.*, $\mathbf{u} = (u, 0)$. The Thiele equation can be expressed explicitly as

$$-F_x^{pin} - Gv_y + Dv_x\alpha - Du_x\beta = 0 \tag{4}$$

$$-F_y^{pin} - F_y - Gu_x + Gv_x + Dv_y\alpha = 0, \tag{5}$$

where F_y originates from the potential $U = U(\mathrm{Y})$ and is essential for skyrmion motion along the x-direction. If we ignore the pinning forces, we obtain the velocity of the steady motion of the skyrmion, which reads.

$$v_x = \frac{\beta}{\alpha}u. \tag{6}$$

Therefore, β/α governs the skyrmion velocity.

Supplementary Note II – Analytical descriptions for skyrmion motion and skyrmion inertia on the track

For a one-dimensional nanotrack, the twist at the edges imposes an extra potential for the skyrmion. As a reasonable approximation, we consider a quadratic potential $U(Y) = \frac{1}{2}kY^2$, where Y is the *y*-component of the skyrmion's center. Ignoring the influence of disorder, we obtain

$$Y(t) = \frac{Gu(\alpha-\beta)}{\alpha k}\left(1 - e^{-\frac{t}{\tau}}\right) \tag{7}$$

$$v_x = u[\frac{\beta}{\alpha} + (1 - \frac{\beta}{\alpha})\frac{G^2 e^{-\frac{t}{\tau}}}{G^2+D^2\alpha^2}] \tag{8}$$

$$X(t) = u[\frac{\beta}{\alpha}t + (1 - \frac{\beta}{\alpha})\frac{G^2\tau}{G^2+D^2\alpha^2}(1 - e^{-\frac{t}{\tau}})], \tag{9}$$

where $\tau = \frac{G^2+D^2\alpha^2}{D\,k\,\alpha}$ is the characteristic time, and $R = (X(t), Y(t))$ describe the skyrmion position. For a DC current, the skyrmion velocity will increase to the enhanced steady speed in Eq. (6) and the y-component of the skyrmion center will saturate eventually.

It is worth mentioning that the stable moving region for the skyrmion in a narrow track is limited, which requires that $|Y(t)| < Y_c/2$, where Y_c is the width of the stable moving region and a rough estimate gives $Y_c{\sim}40\ nm$ for our 100-nm-wide track. It can be seen from Eq. (7) that either a low current density *u* or a short current pulse is sufficient for stable skyrmion motion. Therefore, the critical pulse width w_c, beyond which the skyrmion will be eliminated can be obtained:

$$w_c = -\tau \ln[1 - \frac{Y_c}{2}\frac{\alpha k}{Gu|\alpha-\beta|}], \tag{10}$$

The critical pulse width is direct related to the current density u. For a sufficiently low current density $u < \frac{Y_c}{2}\frac{\alpha k}{G|\alpha-\beta|}$, the skyrmion can move steadily even for a DC current. After switching off the current pulse, the y-component of the skyrmion will decrease to zero again due to the edge repulsion and its *x*-coordinate will continue to increase in this process:

$$Y(t) = Y_w\, e^{-\frac{t-w}{\tau}} \tag{11}$$

$$v_x = -\frac{G\,Y_0}{D\,\alpha\,\tau} e^{-\frac{t-w}{\tau}} \tag{12}$$

$$X(t) = X_w - \frac{G\,Y_0}{D\,\alpha}(1 - e^{-\frac{t-w}{\tau}}) \ , \tag{13}$$

where $Y_w = Y(w)$ and w is the pulse width. For the situation when $\beta > \alpha$, v_x is always positive for both $Q = \pm 1$. The displacement due to inertia after switching off the pulse is given by the expression

$$\Delta X = -\frac{GY_0}{D\alpha} = \frac{G^2 u\,(\beta-\alpha)}{D\,\alpha^2\,k}\left(1 - e^{-\frac{w}{\tau}}\right). \tag{15}$$

Interestingly, if the velocity under the current pulse is defined as $\bar{v} = \frac{X_w + \Delta X}{\mathrm{w}}$, we find that $\bar{v} = \frac{\beta}{\alpha} u$, which is the same as the skyrmion velocity under a DC current.

Supplementary Fig. 9a shows the potential obtained using the micromagnetic simulations for a 100-nm-wide nanotrack for an external field of $H = 100$ mT. The potential is fitted using a quadratic function $U(Y) = \frac{1}{2}kY^2$, with a fitted value of $k = 25.6\ \mathrm{ns}^{-1}$. For typical parameters for skyrmions $\mathrm{G} = 4\pi$, $\mathrm{D} = 4\pi \times 1.2, \alpha = 0.0167$, the characteristic time is established to be $\tau =$ 24.5 ns. Supplementary Fig. 9b shows the skyrmion displacement under a current pulse based on Eqs. (9-15), indicating that the skyrmion continues to move under inertia after switching off the current pulse. Meanwhile, the y-component of the skyrmion takes a relatively small value of ~ 5 nm under the current pulse and then decreases to zero after switching off the pulse due to the edge repulsion, as shown in Supplementary Fig. 9c. The critical pulse width w_c calculated using Eq. (11) is shown in Supplementary Fig. 9d, beyond which the skyrmion will be erased at the edge. These results are consistent with the main text's micromagnetic simulations in Fig. 3.

Supplementary Note III – Estimate of information-density in the 100-nm-wide nanotrack

It is possible to give a rough estimate of the areal density disregarding the influence of pinning effects. Based on the skyrmion-skyrmion interactions described before[7], the safe skyrmion-skyrmion spacing would likely need to exceed 200 nm in FeGe. This roughly corresponds to a maximum information-density of approximately 4Gb/inch^2 in a 100-nm-wide nanotrack. The density can be further increased by selecting materials with smaller skyrmion sizes. The minimum spacing is closely related to the size of skyrmion. For materials with skyrmion sizes around several nanometers, the density can be increased significantly. The position of skyrmion can also be controlled by external means such as artificial pinning sites (e.g., patterning notches along the edges). Therefore, the minimum skyrmion-skyrmion spacing to avoid the non-synchronous motion may be further reduced.

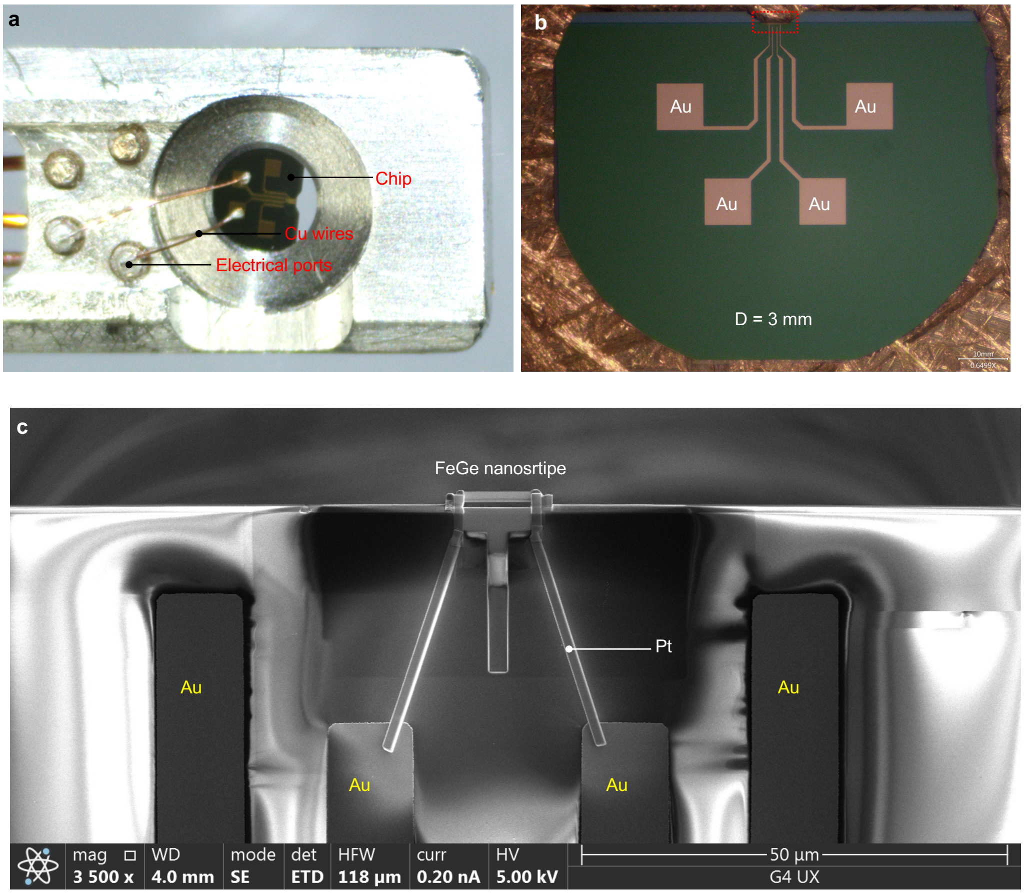

Supplementary Fig. 1 | In-situ electrical Lorentz TEM experimental setup of FeGe micro-device. a, Optical image of the end of a Gatan TEM specimen holder, which was designed for *in situ* electrical biasing and cooling experiments. The four electrical ports were designed by Gatan to be connected to an external voltage source. In order to build the circuit between the ports and the sample, a customized electrical chip with four Au electrodes was self-designed, as shown in **b**. The Au pads were connected to the ports using Cu wires, which were manually fixed using silver colloidal paste (only two ports were used in our experiments). The electrical TEM FeGe micro-device was fabricated using a FIB workstation, as such a chip makes the fabrication process compatible with the conventional FIB lift-out method. **c,** Low magnification SEM image of the FeGe electrical device used for *in situ* Lorentz TEM experiments. The FeGe nanostripe was covered by amorphous carbon on the upper and lower layers. The left and right edges were connected using two Pt electrodes, which were in turn connected to the source of electrical current pulses through the Au electrodes.

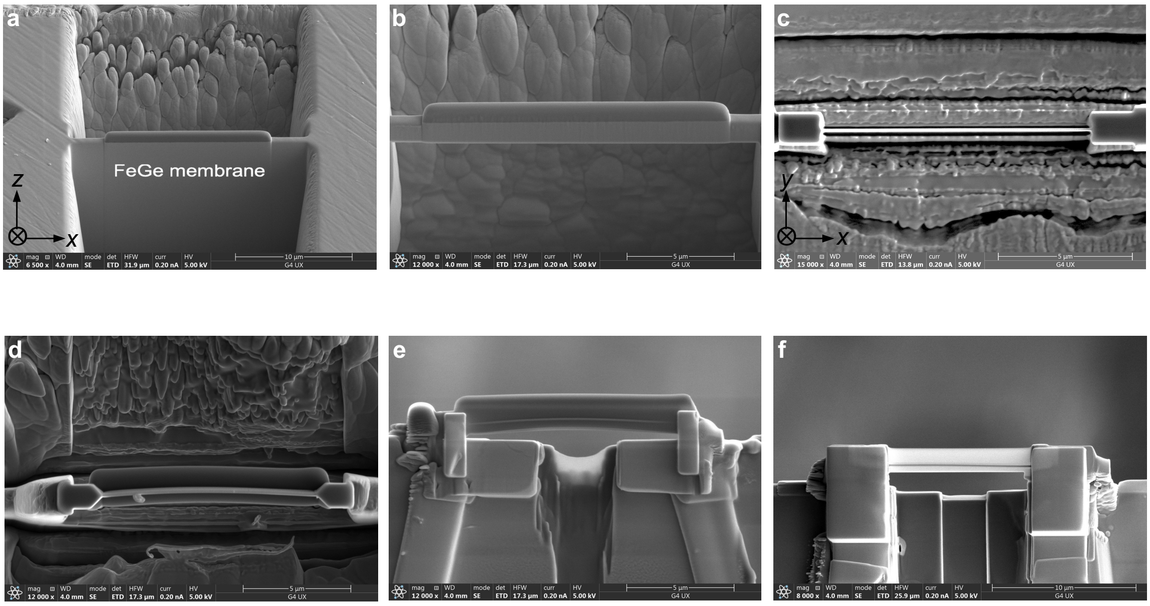

Supplementary Fig. 2 | Procedure for the fabrication of a 100-nm-wide FeGe nanostripe for *in situ* Lorentz TEM using a FIB-SEM dual-beam system. a, An FeGe membrane was carved on the surface of an FeGe single crystal after milling two trenches on each side of a carbon protection layer. **b,** The height of the central membrane was milled down to ~1.2 μm by cutting off the lower part. **c,** Top view of the central membrane after thinning to a thickness of 100 nm, which was the desired width of the nanostripe. The surfaces of the membrane were polished using a small beam current to reduce the amorphous/damaged layer. **d,** The 100-nm-thick nanostripe was protected by carbon layers deposited on each surface. **e,** The carbon-encapsulated membrane was rotated by 90° and fixed onto a customized electrical chip. The left and right ends of the nanostripe were connected to Au electrodes on the chip by fabricating Pt nanosticks. **f,** The FeGe nanostripe was thinned to ~150 nm and the surfaces were polished for TEM observation.

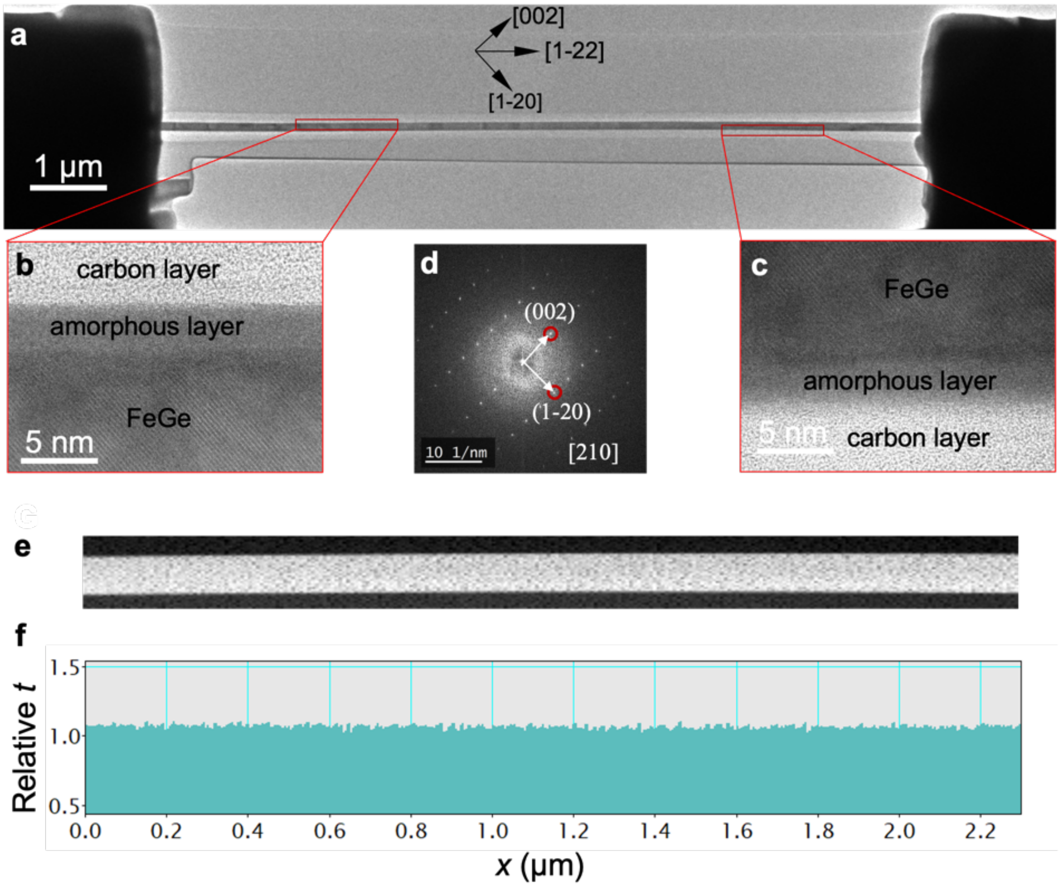

Supplementary Fig. 3 | Characterization of the FeGe nanostripe sample. a, Low magnification TEM image of the FeGe nanostripe. **b,c,** High-resolution TEM images of the upper and lower interfaces between the FeGe nanostripe and the carbon layers. The amorphous layer has a thickness of ~3 nm. **d,** The fast Fourier transformation (FFT) of the TEM image in **c**. The diffraction reflections and crystallographic orientation are indicated in **a** and **d**, respectively. **e,** HAADF-STEM image of the FeGe nanostripe. **f,** Corresponding relative thickness map of the FeGe nanostripe measured using low-loss EELS. The relative thickness (t) profile is plotted with the horizontal direction along x axis. The high-angle annular dark-field (HAADF) STEM, high-resolution TEM, energy-dispersive X-ray (EDX) and electron energy-loss spectroscopy (EELS) were carried out at 300 kV using a Thermo Fisher Themis Z microscope equipped with a field-emission electron gun, a DCOR probe corrector, a DCOR image corrector, a super-X EDX detector and a Gatan Continuum EELS system. EDX maps were recorded and analyzed using Velox software.

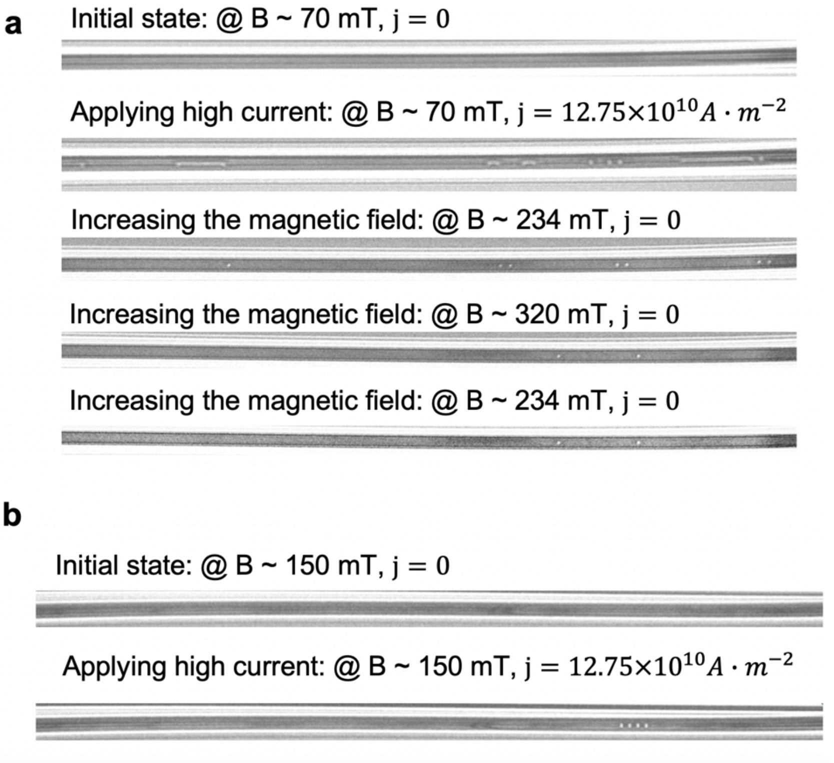

Supplementary Fig. 4 | Creation and isolation of skyrmions in the FeGe nanostripe. The skyrmions were generated through Joule heating effects by applying a higher current under a certain magnetic field. The detailed procedures for generating and isolating the skyrmions are described as follows: (1) For single or two skyrmions, they are generated and isolated in the same way as shown in Supplementary Fig. 4a. Initially, the sample was saturated with a very high magnetic field, and then the field was reduced to ~ 70 mT to maintain the conical state. Subsequently, an electrical current with a high current density of j = 12.75 × 10^{10} $A \cdot m^{-2}$ and a pulse duration of 5 ns was applied. This process created skyrmions due to Joule heating effects. The current was then turned off and the magnetic field was increased to stabilize the skyrmions. To further reduce the number of skyrmions in the nanostripe, the magnetic field was increased gradually, systematically annihilating skyrmions one by one until only two or a single skyrmion remained. Once this configuration was achieved, the magnetic field was decreased to ~234 mT for the current-driven dynamics. This method is straightforward and robust, allowing us to consistently create single skyrmion or two skyrmions in the nanostripe. (2) While for generating skyrmion chains with varying numbers, the process involves some level of probability. The sample was initialized set to the conical state,

similar to the method used for single or two skyrmions above. However, in this case, the magnetic field is maintained at a relatively higher value of ~150 mT. This higher magnetic field is chosen to prevent the creation of too many skyrmions by the thermal effects. By applying a high current density of $j = 12.75 \times 10^{10}\ A \cdot m^{-2}$, skyrmion chains can be directly generated with a certain probability as shown in Supplementary Fig. 4b with a skyrmion number of 4. However, it should be noted that we cannot control the exact number of skyrmions in the chains in a deterministic manner. This method allows for the creation of varying numbers of skyrmions, but the specific quantity in each chain is subject to variability inherent to the process.

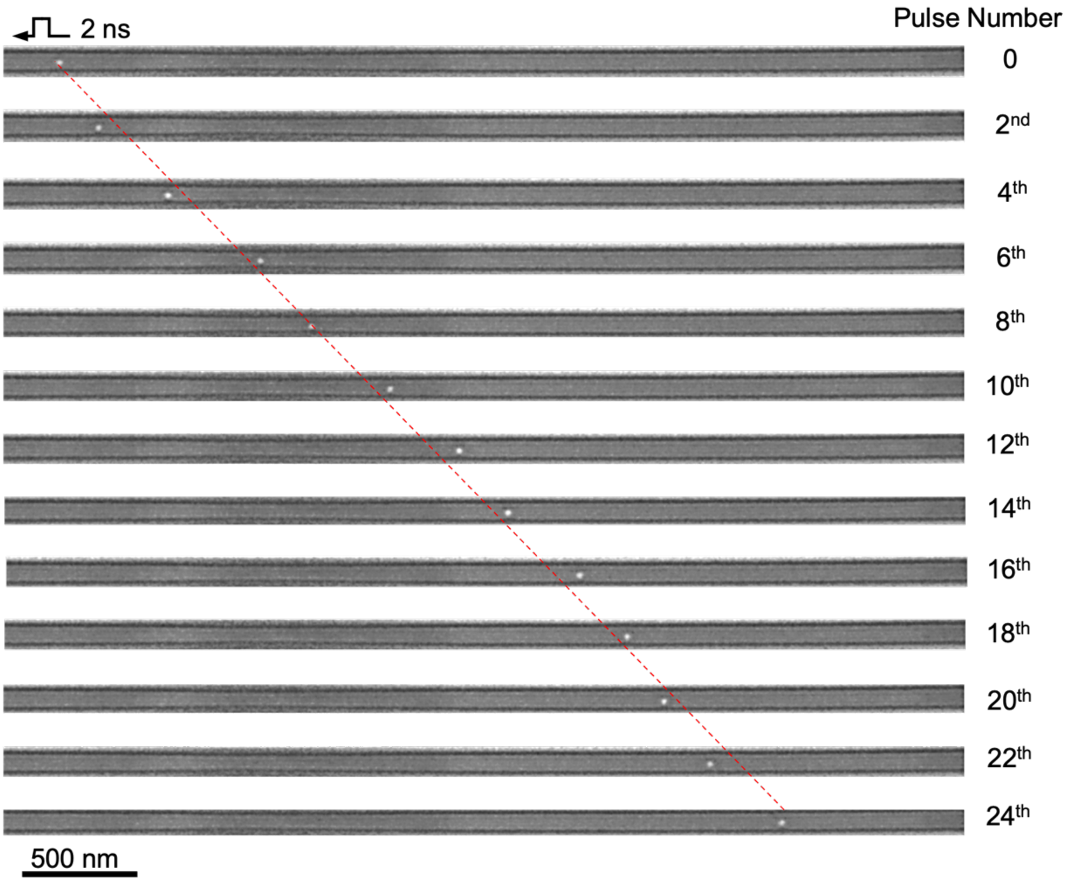

Supplementary Fig. 5 | Motion of a single skyrmion in the FeGe nanostripe using a pulse duration of 2 ns. Representative snapshots of single skyrmion motion at successive current pulses. The trajectory of the single skyrmion is guided by the red dashed line. The current density is $j = 11.5 \times 10^{10} A \cdot m^{-2}$.

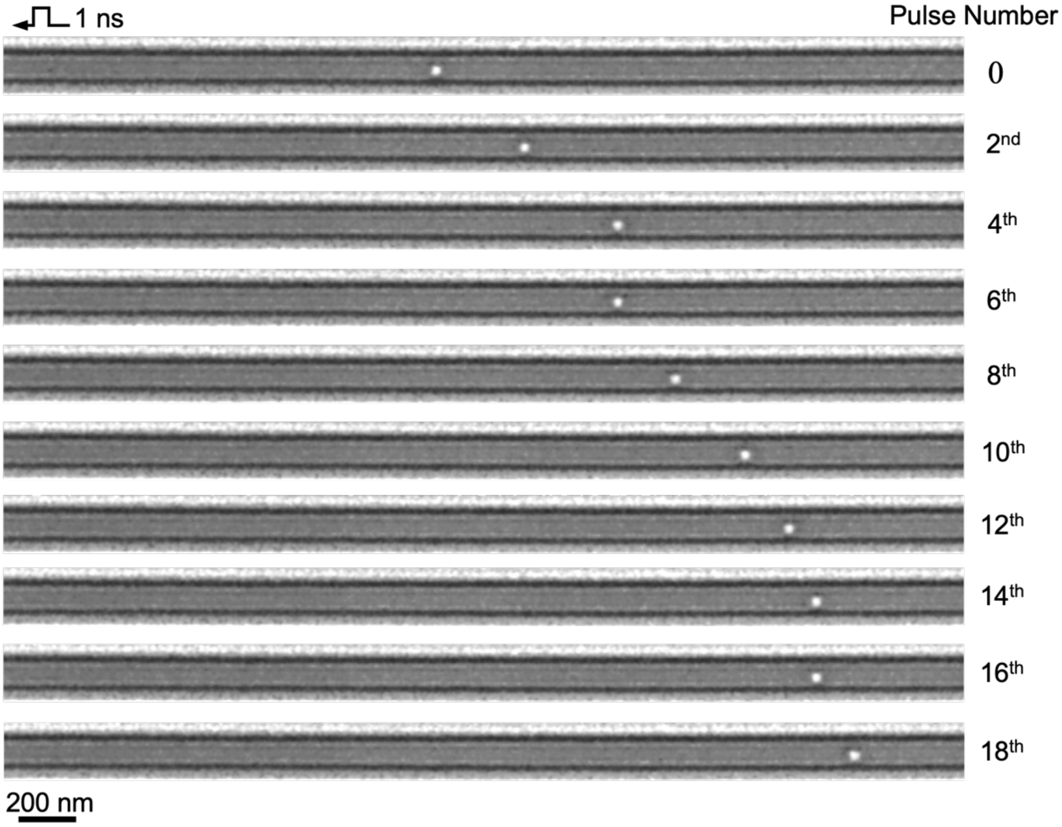

Supplementary Fig. 6 | Motion of a single skyrmion in the FeGe nanostripe using a pulse duration of 1 ns. Representative snapshots of single skyrmion motion using successive current pulses. The single skyrmion is pinned very easily, even at a high current density. The current density is $j = 19.9 \times 10^{10} A \cdot m^{-2}$.

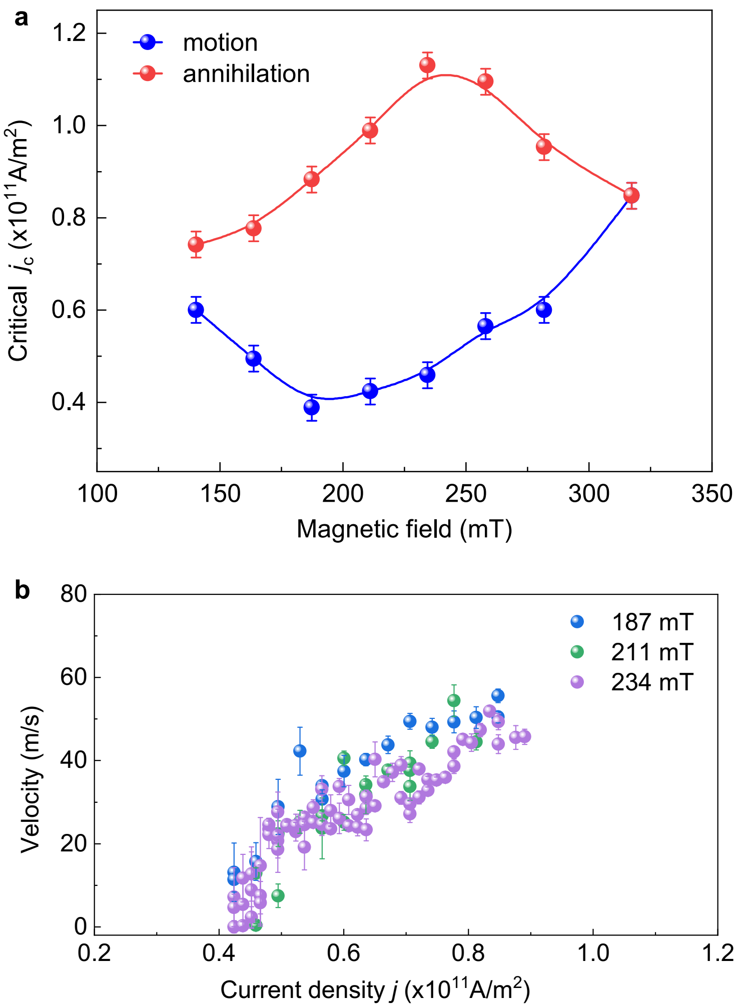

Supplementary Fig. 7 | Skyrmion motion under varied magnetic fields. a, The lower (motion) and upper (annihilation) critical current density with a pulse duration of 5 ns as a function of magnetic field. The lower critical current density corresponds to the threshold at which the skyrmion begins to move, while the upper critical current density is the point at which the skyrmion is annihilated upon application of the current pulse. **b,** Skyrmion velocities plotted as a function of current density under different magnetic fields. The pulse duration is 5 ns.

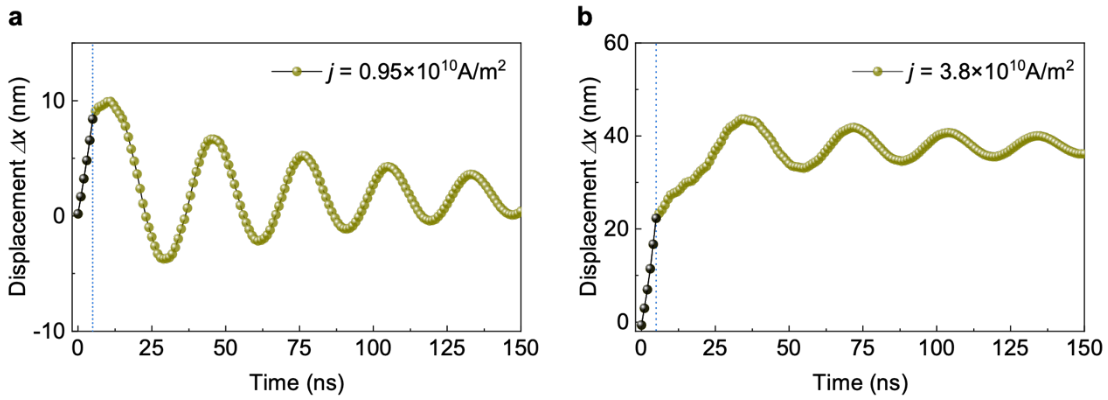

Supplementary Fig. 8 | Micromagnetic simulations of skyrmion inertia in a confined FeGe nanostripe. Typical skyrmion displacement ΔX as a function of time after applying a current pulse duration of 5 ns at various current densities, with the consideration of pinning effects. The vertical dashed lines indicate a time of 5 ns.

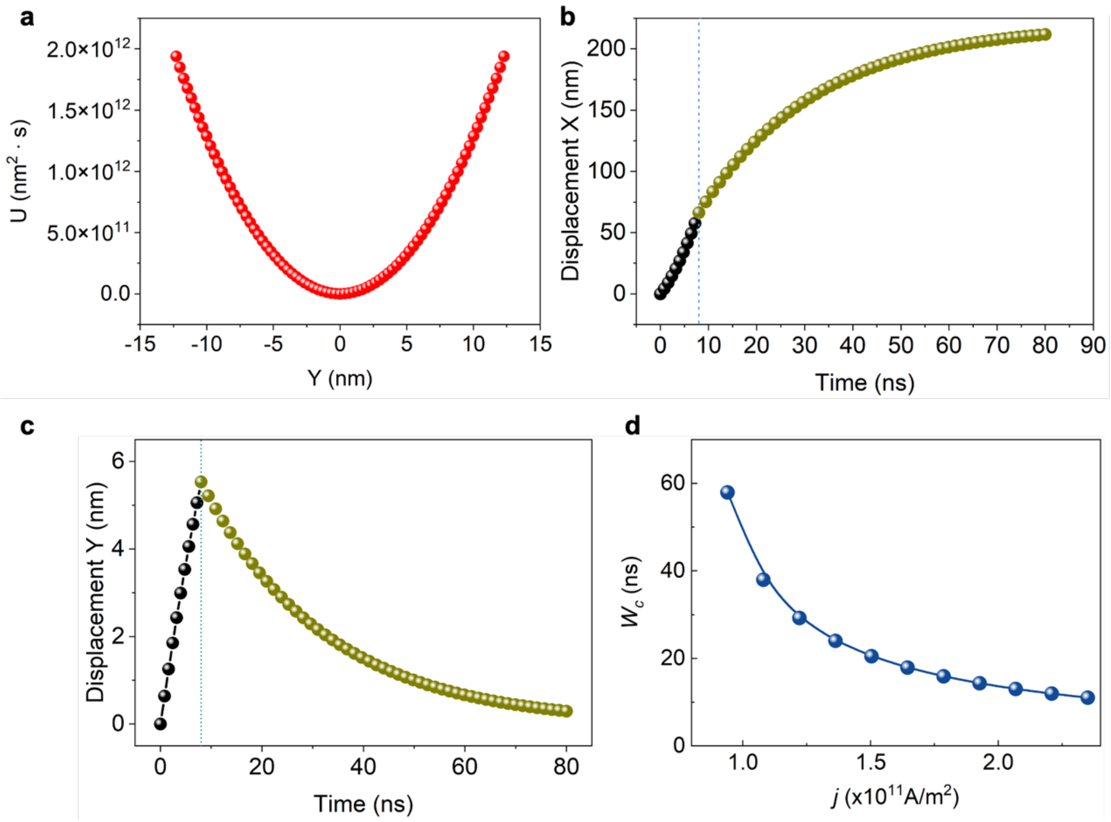

Supplementary Fig. 9 | Skyrmion inertia in the confined FeGe nanostripe. **a,** Quadratic potential of the skyrmion in the track. The red dots represent the data extracted from micromagnetic simulations. **b,** Skyrmion x-component displacement under a current pulse plotted using Eqs. (10) and (14) in Supplementary Note II. **c,** y-component of the skyrmion displacement under the same current pulse. **d,** Critical pulse width w_c, beyond which the skyrmion will be erased at the edge, calculated using Eq. (11) in Supplementary Note II. The parameters are: pulse width 8 ns, current density $j \sim 4.7\times10^{10}$ A/m^2, $\mathrm{G} = 4\pi$, $\mathrm{D} = 4\pi \times 1.2$, $k = 25.6\ \mathrm{ns}^{-1}$, $\alpha = 0.0167$ and $\beta = 0.1336$. The dark yellow dots denote the skyrmion motion under inertia after switching off the current pulse.

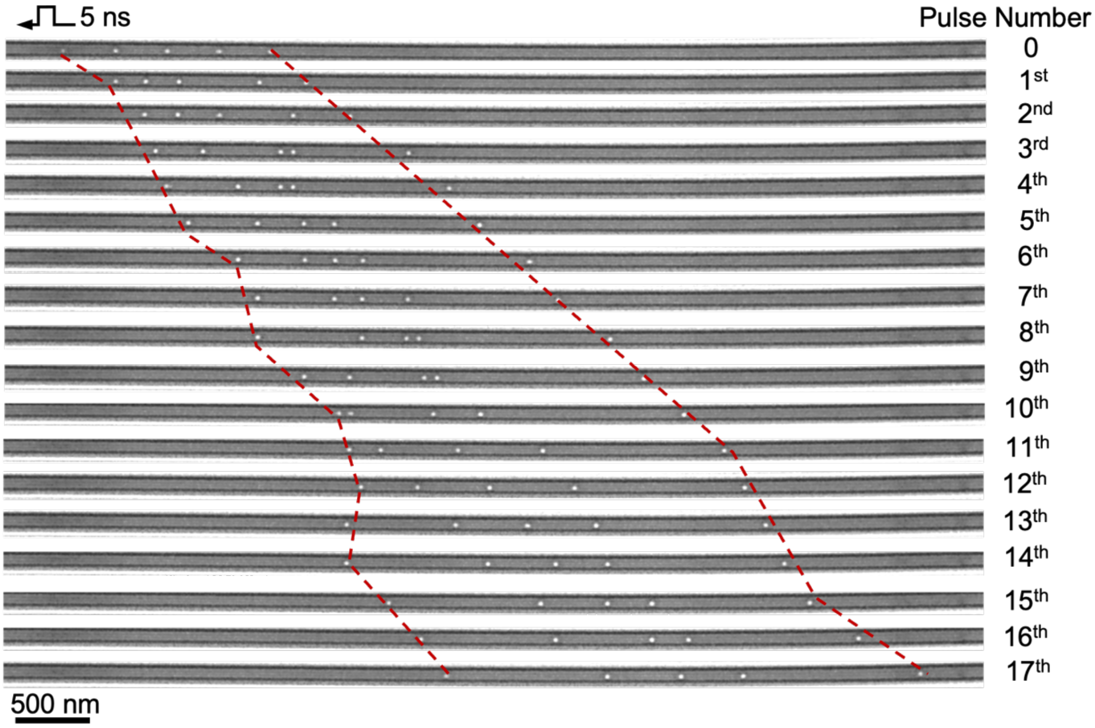

Supplementary Fig. 10 | Collective motion of a skyrmion chain in the FeGe nanostripe. Representative snapshots of skyrmion motion at successive current pulses. The trajectories of the first and last skyrmion are guided by dashed red lines. The current density is $j = 10.1 \times 10^{10} A \cdot m^{-2}$ and the pulse duration is 5 ns.

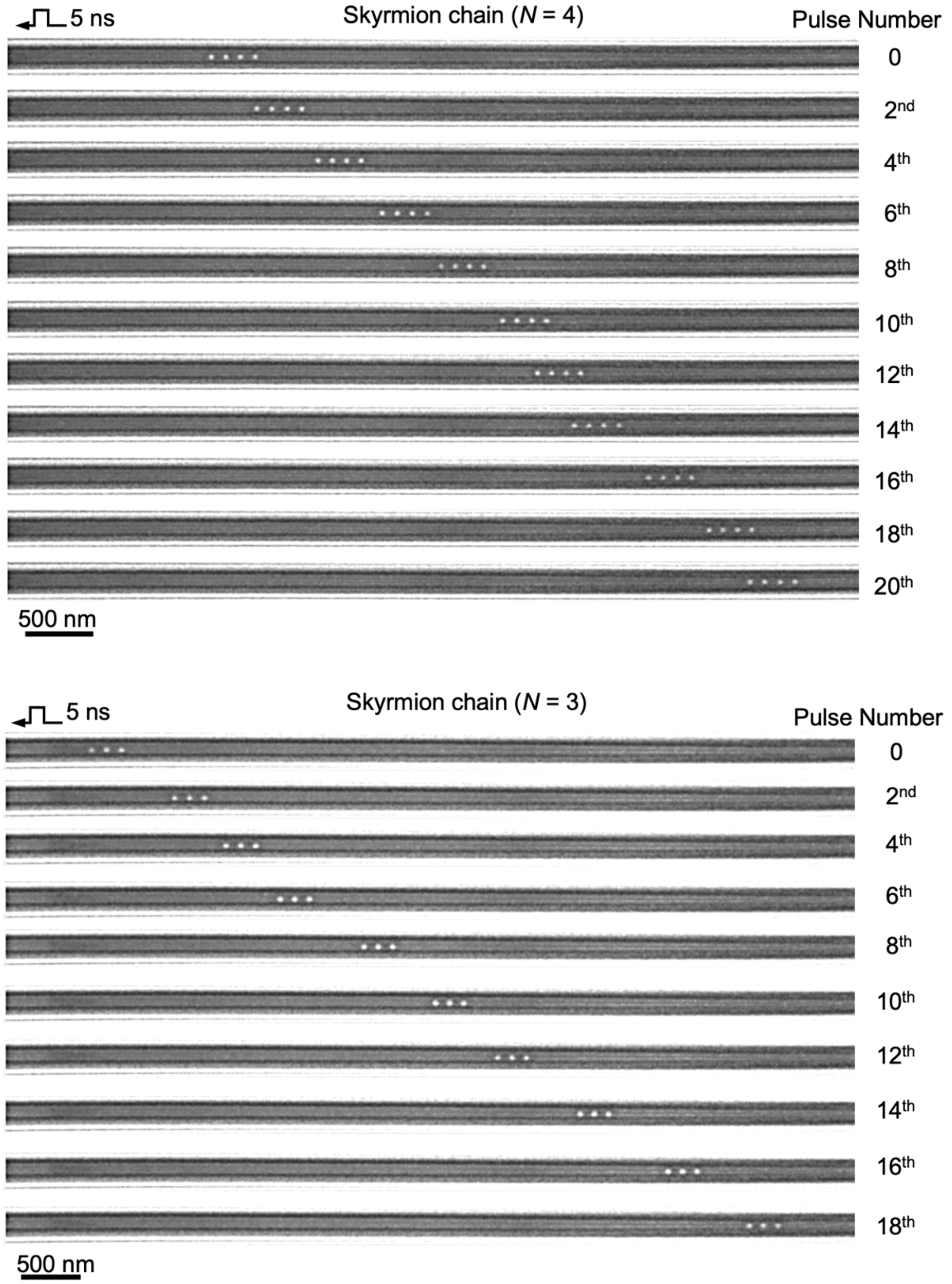

Supplementary Fig. 11 | Motion of skyrmion chains (with $N = 4$ and 3) in the FeGe nanostripe. Representative snapshots of skyrmion motion at successive current pulses. The current density is $j = 7.75 \times 10^{10} A \cdot m^{-2}$ and $j = 8.47 \times 10^{10} A \cdot m^{-2}$, respectively. The pulse duration is 5 ns.

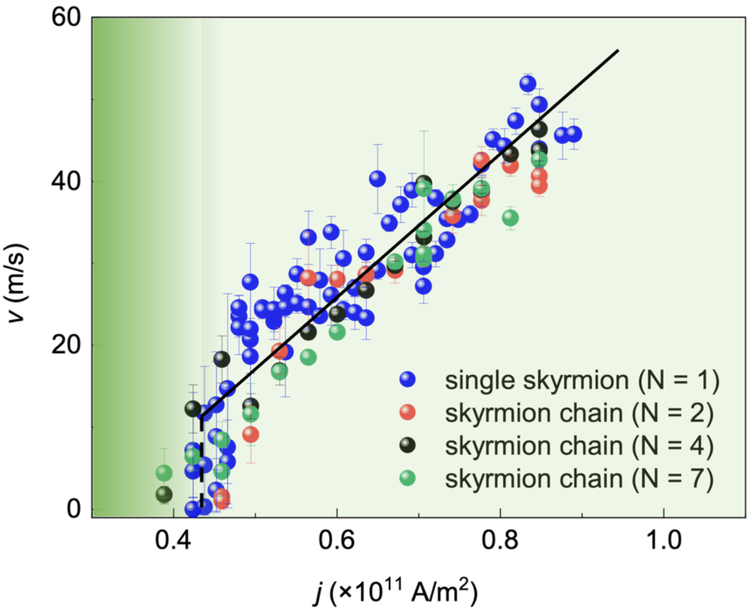

Supplementary Fig. 12 | Skyrmion velocities plotted as a function of current density. The skyrmion chains with various skyrmion number are plotted together for comparison. The pulse duration is 5 ns.

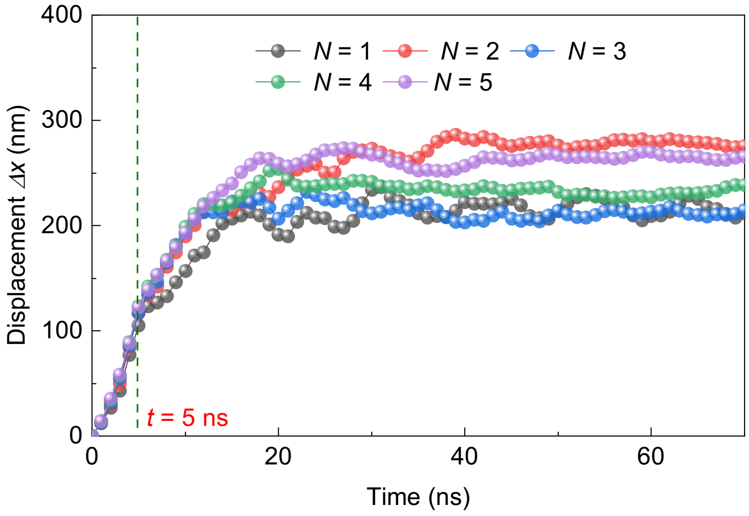

Supplementary Fig. 13 | Skyrmion displacement ΔX plotted as a function of time for skyrmion chains with various skyrmion number (*N*). The pulse duration is 5 ns and the current density is $j = 10\times10^{10}$ A/m^2. The vertical dashed line indicates a time of 5 ns. The variations in displacements are caused by the randomly distributed pinning sites modeled in the nanostripe.

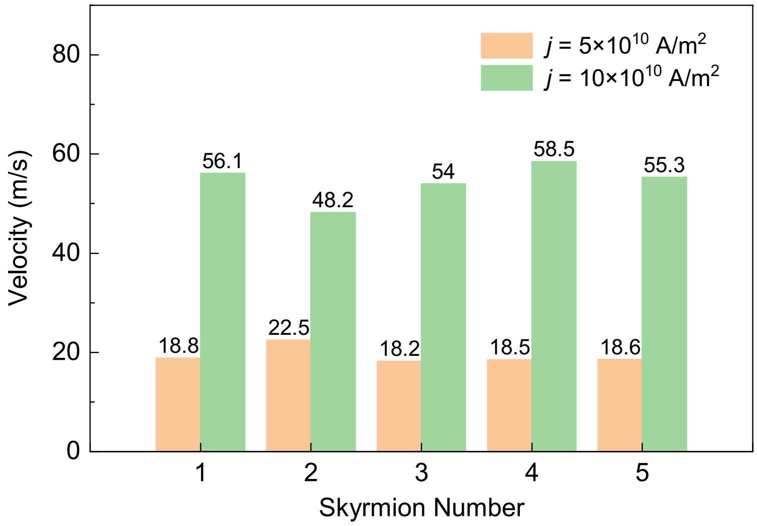

Supplementary Fig. 14 | Simulated velocities of skyrmion chains with a various number of skyrmion. The current densities are 5×10^{10}A/m^2 (orange) and 10×10^{10}A/m^2 (green), respectively. The pulse duration is 5 ns. The velocities are averaged over five current pulses in the simulations. The variations are primarily due to pinning effects and the limited number of current pulses applied in the simulations.

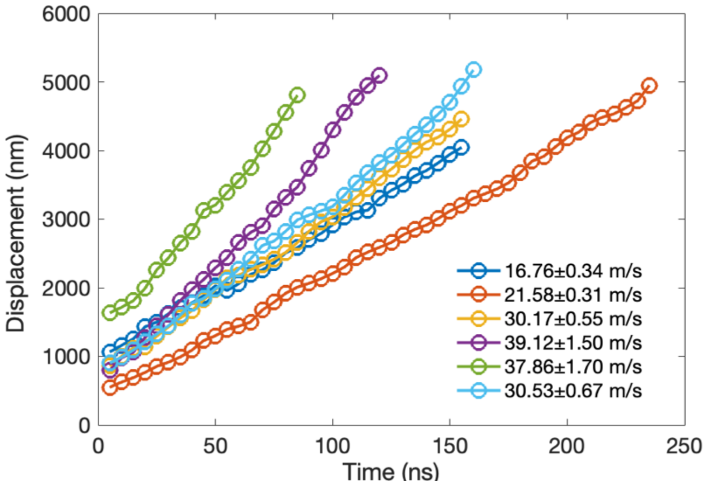

Supplementary Fig. 15 | Skyrmion displacement as a function of pulse time. The lines in different colors correspond to the skyrmion motion under varied current densities. The pulse duration is 5 ns here.

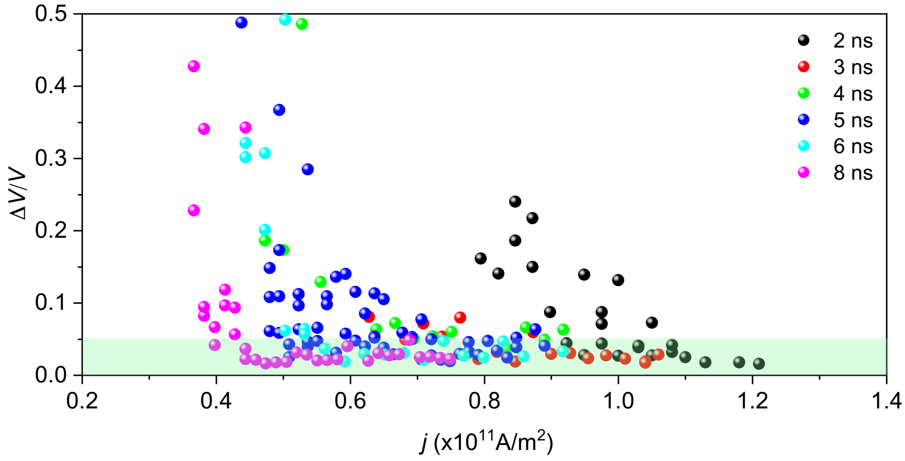

Supplementary Fig. 16 | The ratio of skyrmion velocities (v) and their standard deviations (Δv) under various current densities and pulse durations. Values below 5% are highlighted in the green box for reference.

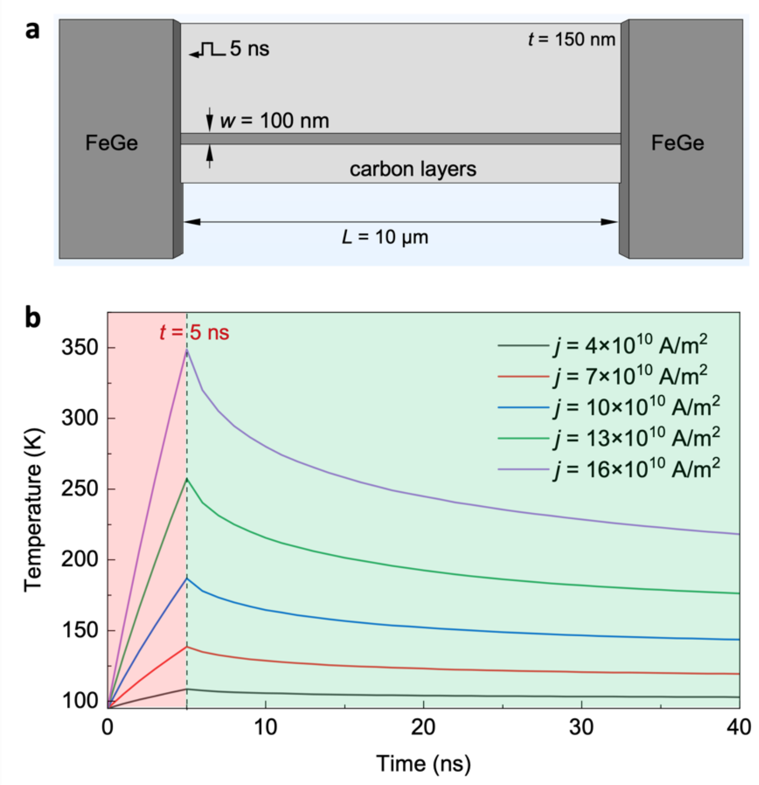

Supplementary Fig. 17 | Simulated local heating effects in a 100-nm-wide FeGe nanostripe using COMSOL Multiphysics software. a, Schematic of the device geometry for simulation. **b,** Temperature profiles are plotted over time. The pulse duration is 5 ns. The vertical dotted line indicates the time at 5 ns, after which the current is turned off.

References

1. Zhang, S. & Li, Z. Roles of Nonequilibrium Conduction Electrons on the Magnetization Dynamics of Ferromagnets. *Phys. Rev. Lett.* **93**, 127204 (2004).
2. Thiaville, A., Nakatani, Y., Miltat, J. & Suzuki, Y. Micromagnetic understanding of current-driven domain wall motion in patterned nanowires. *Europhys. Lett. EPL* **69**, 990–996 (2005).
3. Zang, J., Mostovoy, M., Han, J. H. & Nagaosa, N. Dynamics of Skyrmion Crystals in Metallic Thin Films. *Phys. Rev. Lett.* **107**, 136804 (2011).
4. Iwasaki, J., Mochizuki, M. & Nagaosa, N. Universal current-velocity relation of skyrmion motion in chiral magnets. *Nat. Commun.* **4**, 1463 (2013).
5. Nagaosa, N. & Tokura, Y. Topological properties and dynamics of magnetic skyrmions. *Nat. Nanotechnol.* **8**, 899–911 (2013).
6. Everschor, K. *et al.* Rotating skyrmion lattices by spin torques and field or temperature gradients. *Phys. Rev. B* **86**, 054432 (2012).
7. Du, H. *et al.* Interaction of Individual Skyrmions in a Nanostructured Cubic Chiral Magnet. *Phys. Rev. Lett.* **120**, 197203 (2018).
