# Supplementary material for: Steady motion of 80-nm-size skyrmions in a 100-nm-wide track
Source: Nat Commun. 2024 Jul 4;15:5614. doi: 10.1038/s41467-024-49976-6 (PMC11224351; doi:10.1038/s41467-024-49976-6)
Supplement: Supplementary file 3 — Description of Additional Supplementary Files [file 41467_2024_49976_MOESM3_ESM.pdf]

Description of Additional Supplementary Files

Supplementary Movie 1
Motion of a single skyrmion under a pulse duration of 5 ns and a current density of $8.47 \times 10^{10} A \cdot m^{-2}$ in the 100-nm-wide track.

Supplementary Movie 2
Reversed motion of a single skyrmion under a pulse duration of 5 ns and a current density of -$8.47 \times 10^{10} A \cdot m^{-2}$ in the 100-nm-wide track.

Supplementary Movie 3
Annihilation of a single skyrmion under a pulse duration of 5 ns and a current density of $11.0 \times 10^{10} A \cdot m^{-2}$ in the 100-nm-wide track.

Supplementary Movie 4
Motion of a single skyrmion under a pulse duration of 2 ns and a current density of $11.5 \times 10^{10} A \cdot m^{-2}$ in the 100-nm-wide track.

Supplementary Movie 5
Motion of a single skyrmion under a pulse duration of 1 ns and a current density of $19.9 \times 10^{10} A \cdot m^{-2}$ in the 100-nm-wide track.

Supplementary Movie 6
Simulated dynamics of a single skyrmion under a pulse duration of 5 ns and a current density of $5.7 \times 10^{10} A \cdot m^{-2}$ in the 100-nm-wide track.

Supplementary Movie 7
Skyrmion inertia motion under a pulse duration of 5 ns and a current density of $5.7 \times 10^{10} A \cdot m^{-2}$ in the 100-nm-wide track.

Supplementary Movie 8
Simulated skyrmion annihilation under a long pulse duration in the 100-nm-wide track.

Supplementary Movie 9
Collective motion of two isolated skyrmion under a pulse duration of 5 ns and a current density of $7.4 \times 10^{10} A \cdot m^{-2}$ in the 100-nm-wide track.

Supplementary Movie 10
Collective motion of skyrmion pair under a pulse duration of 5 ns and a current density of $7.4 \times 10^{10} A \cdot m^{-2}$ in the 100-nm-wide track.

Supplementary Movie 11
Collective motion of skyrmion chain under a pulse duration of 5 ns and a current density of $7.4 \times 10^{10} A \cdot m^{-2}$ in the 100-nm-wide track.
